# Relationship between cumulative silica exposure and silicosis: a systematic review and dose-response meta-analysis

**DOI:** 10.1136/thorax-2024-221447

**Published:** 2024-08-06

**Authors:** Patrick Howlett, Jeffrey Gan, Maia Lesosky, Johanna Feary

**Affiliations:** 1 National Heart and Lung Institute, Imperial College London, London, UK; 2 Department of Occupational Lung Disease, Royal Brompton Hospital, London, UK

**Keywords:** Occupational Lung Disease, Clinical Epidemiology

## Abstract

**ABSTRACT:**

**Background:**

Silicosis, a chronic respiratory disease caused by crystalline silica exposure, is a persistent global lung health issue. No systematic review of the relationship between cumulative respirable crystalline silica (RCS) exposure and silicosis exists. UK exposure limits are currently under review. We therefore performed a systematic review and dose-response meta-analysis of this relationship.

**Methods:**

Web of Science, Medline and Embase were searched on 24 February 2023. Studies of radiographic, autopsy or death certificate silicosis, with an estimated average follow-up of over 20 years since first employment, were included. Cumulative silicosis risk methods were compared. The relative risks (RR) of silicosis at increasing cumulative exposures were calculated and used to estimate the absolute risk reduction (ARR).

**Results:**

Eight eligible studies, including 10 cohorts, contributed 8792 cases of silicosis among 65 977 participants. Substantial differences in cumulative risk estimates between methodologies exist. Using the same method, we observed higher cumulative silicosis risks among mining compared with non-mining cohorts. A reduction from 4 to 2 mg/m³-years in cumulative RCS exposure corresponded to substantial risk reductions among miners (RR 0.23 (95% CI 0.18 to 0.29, I^2^=92.9%) with an ARR of 323 (95% CI 298 to 344) per 1000) and non-miners (RR 0.55 (95% CI 0.36 to 0.83, I^2^=77.0%) with an ARR of 23 (95% CI 9 to 33) per 1000).

**Conclusion:**

Despite significant heterogeneity, our findings support a reduction in permissible exposure limits from 0.1 mg/m^3^ to 0.05 mg/m³, particularly among mining populations. Further research is needed among non-miners as only two studies were eligible.

**WHAT IS ALREADY KNOWN ON THIS TOPIC**

- Despite decades of research, evidence regarding the relationship between cumulative respirable silica and silicosis has not been appraised in a systematic review process or quantified using meta-analysis methods.

**WHAT THIS STUDY ADDS**

- Using reproducible methods, we showed substantial differences in cumulative risk estimates of individual studies, dependent on which methodology was used. In a dose-response meta-analysis, we demonstrated clinically important reductions in silicosis risk at cumulative respirable silica thresholds relevant to UK and US policy debates. Silicosis risks appear different among miners than non-miners, although only two studies of non-miners were included.

**HOW THIS STUDY MIGHT AFFECT RESEARCH, PRACTICE OR POLICY**

- There is current debate regarding respirable crystalline silica exposure limits in the UK and recent debate among miners in the USA. This research supports the reduction of permissible exposure limits from 0.1 mg/m^3^ to 0.05 mg/m^3^. More studies of non-miners are needed.

## Background

 Silicosis is an incurable respiratory disease caused by exposure to respirable crystalline silica (RCS). The global burden of silicosis is unclear; high prevalence among mining^1^ and non-mining industries,^2^ ongoing high exposures and concerns of under-reporting mean silicosis morbidity and mortality is likely far higher than modelled estimates.^3^ Chronic silicosis, the most common form, may present decades after lower dose exposures and is considered the primary outcome of this study. Acute and accelerated silicosis presents within 10 years of high intensity exposure. Lower grade silicosis may be asymptomatic, but disease progression after exposure cessation can occur with subsequent disabling dyspnoea and cough. Silica exposure is also associated, in a dose-response manner, with silicosis mortality, lung cancer, tuberculosis, autoimmune disease and renal disease.^4 5^

Despite decades of research, permissible exposure limits (PELs)—representing average intensity of RCS exposure over an 8-hour working shift—are debated and range from 0.05 (USA) to 0.35 mg/m^3^ (China).^6^ The most comprehensive review of silica exposure risks was the 2016 US Occupational Safety and Health Administration (OSHA) Federal ruling, resulting in reduction of the US PEL from 0.1 mg/m^3^ to 0.05 mg/m^3^.^7^ The OSHA’s silicosis prevalence estimates were 60–773/1000 workers for 45- year exposures at an average exposure of 0.1 mg/m^3^ and 20–170/1000 workers for 45- year exposures at 0.05 mg/m^3^.^7^ The OSHA legislation excludes the US mining industry in which the PEL was reduced from 0.1 mg/m^3^ to 0.05 mg/m^3^ earlier in 2024.^8^ There is a current UK government call for evidence regarding this same reduction (0.1 mg/m^3^ to 0.05 mg/m^3^).^9^

The OSHA review findings should be interpreted cautiously. First, the review methods are not described. Second, the prevalence estimates used relied on respective study methods and in one case were estimated from graphs.^10^ Finally, the authors compared different outcomes—for example, modelled (instantaneous) odds of disease^11^ and cumulative risk.

The OSHA conclusions were similar to those of a prior review which stated that “30 years exposure at 0.1 mg/m^3^ might lead to a lifetime silicosis risk of about 25%, whereas reduction of the exposure to 0.05 mg/m^3^ might reduce the risk to under 5%“.^12^ Both reviews noted between-study heterogeneity, partly explained by methodological differences. Conversely, plots comparing non-parametric cumulative risks demonstrate similarities between multiple selected studies.^13 14^

A limitation of the above evidence is the use of non-systematic review methods to present narrative findings and, in some cases, non-transparent methods to provide quantitative summaries. Furthermore, no study has investigated the impact of different cumulative risk calculation methods used by included studies. An intrinsic limitation of using radiographic silicosis as an outcome is the presence of sub-radiological silicosis, even at higher autopsy and CT grades of disease.^15^ The recent development of a ‘dose-response meta-analysis’ enables the meta-analysis of relative risks of silicosis from different study methodologies (eg, case-control, cohort study) across RCS dose categories.^16^

We therefore aimed to investigate the relationship between cumulative RCS exposure and silicosis risk by (1) systematically reviewing studies in which adequate latency to achieve the outcome has been achieved; (2) using a life table approach to calculate the cumulative silicosis risk within reported exposure categories; and (3) performing a dose-response meta-analysis to quantify the relationship and heterogeneity between studies.

## Methods

### Search strategy and selection criteria

This review follows the Meta-analyses Of Observational Studies in Epidemiology (MOOSE) statement and was registered with PROSPERO (CRD42023401673). Studies indexed before 24 February 2023 were retrieved from Web of Science, Medline and Embase. Search strategies included terms for ‘silicosis’ and ‘cumulative exposure’ (see online supplemental file 1). All studies in previous reviews were captured by our searches.^7 12^ Selection criteria included English language studies of RCS-exposed adults, reporting an individual estimation of RCS and subsequent categorical dose ranges. Silicosis was defined by radiological diagnosis, using International Labour Organisation (ILO) classification or similar, or autopsy. We included studies where mean or median duration since starting work was estimated at >20 years; this represented a pragmatic balance of capturing the increased silicosis risk observed after 20 years^10 17 18^ while maintaining adequate study numbers.

**Figure 1:**
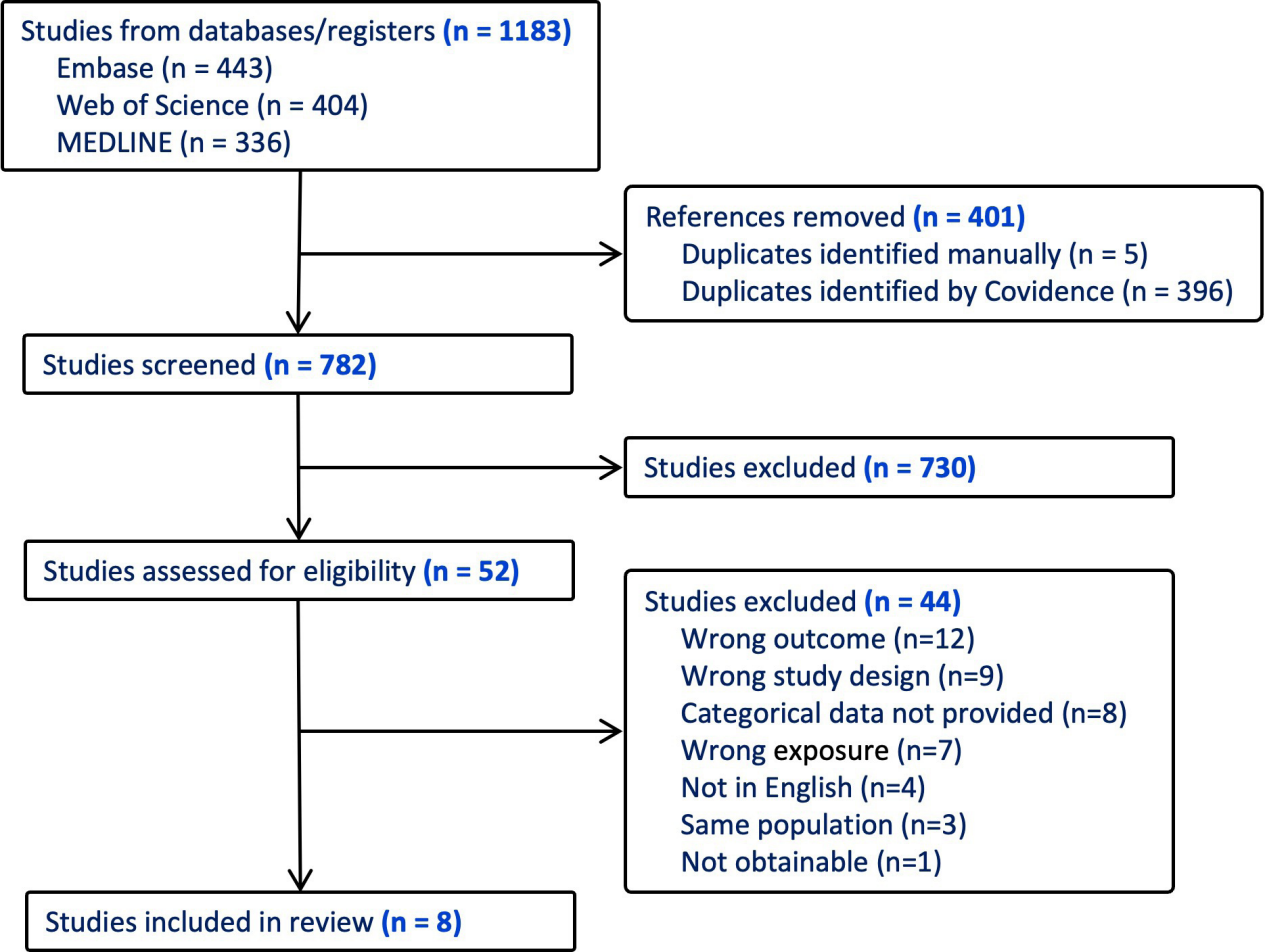
PRISMA diagram.

Study selection and data extraction were performed on the Covidence platform (figure 1). Following de-duplication, two authors (JG, PH) independently screened abstracts and full texts, with conflicts resolved through final author review (JF). Contact with authors of studies with plausible population overlap was attempted but not successful. In these cases, we chose the study with the most detailed exposure categories.

### Data analysis

Two authors (JG, PH) independently extracted study characteristics and performed quality assessment (see online supplemental file 2). Concentrations and categorical cumulative RCS or dust exposures were extracted. Where cumulative dust exposures were presented, we used the study’s own reported conversion equation to estimate RCS.^10 19^ Study quality was assessed using an adapted version of the Newcastle–Ottawa Scale (NOS). We removed the NOS ‘comparability’ section as our relationship of interest was univariate; adjustment for confounding and effect was not relevant.

### Data synthesis

**Figure 2:**
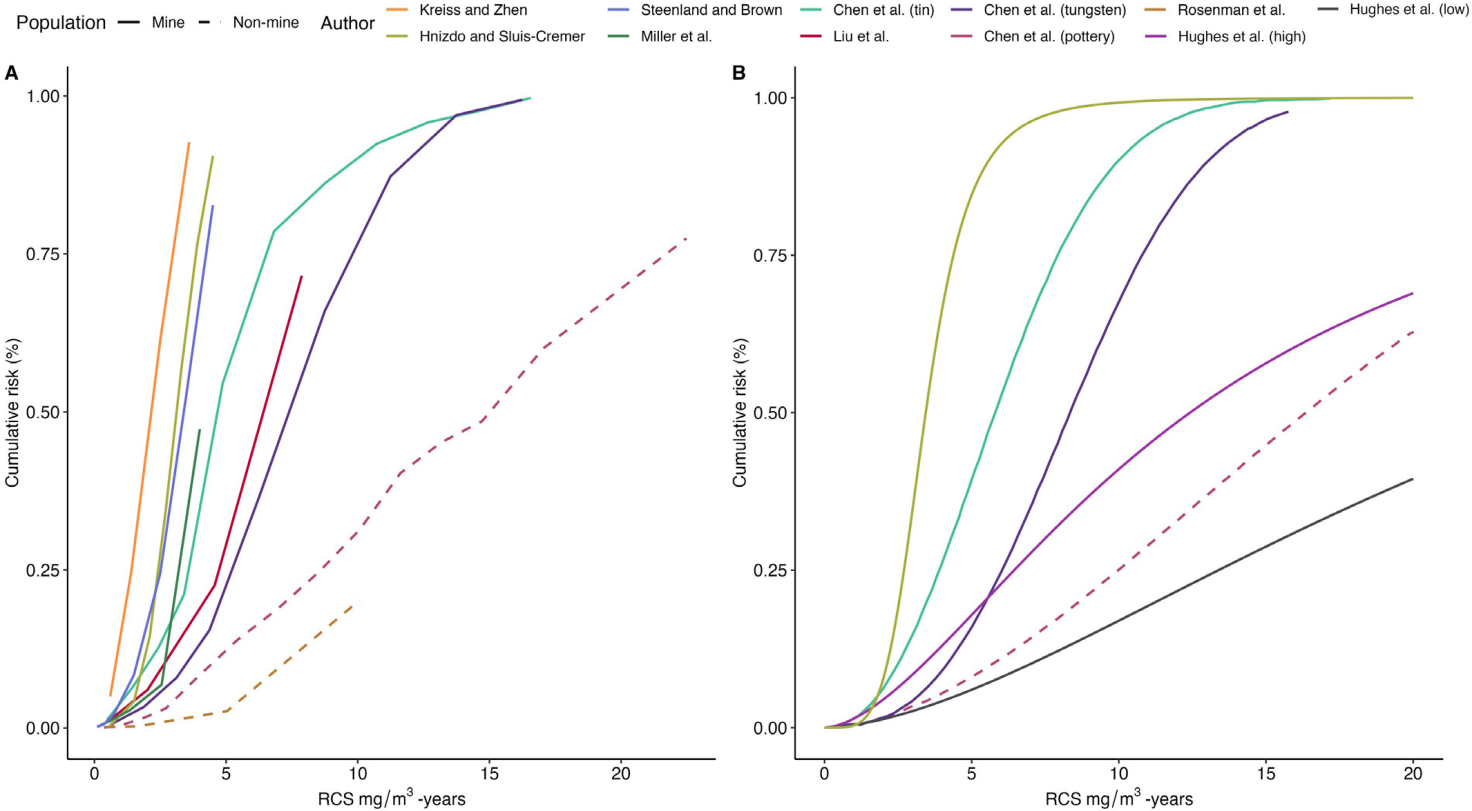
Comparison of cumulative risk of silicosis according to cumulative respirable crystalline silica (RCS) exposure. (A) Life table re-calculated cumulative risk for each study estimated using the Steenland formula. (B) Fitted parametric cumulative risk curve. These were either plotted using the formulae presented in the respective paper or, in the case of Chen *et al*,^10^ as no formulae were provided, plotted using locally estimated least squares regression (LOESS) from a total of 269 paired values across the three cohorts.

For all studies we re-calculated the cumulative risk of silicosis by the SAS LIFETEST method used by Chen *et al* and Hnizdo and Sluis-Cremer,^10 19 20^ and the method used by Steenland and Brown^14^ (see online supplemental file 3). For dose ranges where the lower limit was missing, it was assumed zero. Where the upper limit was missing, it was assumed the highest category width was 1.5× greater than the previous category. We plotted re-calculated cumulative risks and then compared them with the reported and model fitted cumulative risks. For Chen *et al*,^10^ as no coefficients were provided, we used WebPlotDigitizer^21^ automated extraction of fitted cumulative risk values from figure 2B in the article and extracted every 5 pixels providing 269 paired values.

We performed a dose-response meta-analysis using standard parameters of the dosresmeta package^16^ in R (version 4.3.2). The meta-analysis takes log relative risks (including odds ratios (OR) or rate ratios), their confidence intervals (CI) and doses as inputs. Where the number of individuals per category was reported, we calculated the univariate odds of silicosis risk per category, and then the OR for that category compared with the baseline (lowest dose) category. For two studies reporting person-years, incidence rate ratios were calculated using a similar approach.^22 23^ Both OR and rate ratios were converted to log relative risks. CIs were calculated using the delta method. Unless provided, we estimated dose at the midpoint of each category using the methods described above. For Hnizdo and Sluis-Cremer^19^ we used data in a second publication,^24^ as risks were equivalent but more categories were provided. Our descriptive plots showed a differential non-linear response between miners and non-miners. Post-hoc, therefore, we fitted separate restricted cubic spline models for each population. For the miner model (dose groups=55) we chose four knots at default quantiles (0.05, 0.35, 0.65 and 0.95) equivalent to 0.4, 2.0, 3.9 and 12.0 mg/m^3^-years of RCS exposure. For the non-miner model (dose groups=18) we chose three default knots (0.1, 0.5, 0.9) equivalent to 0.9, 6.2 and 15.4 mg/m^3^-years. Departure from linearity was tested using the Wald test. To provide context for the current UK and US debate regarding the change of PELs, we fixed the baseline relative risk at 4 mg/m^3^-years, equivalent to 40 years working at 0.1 mg/m^3^.

We used ORs as a standardised effect measure assuming limited impact of censoring. This was justified as the loss to follow-up among cohort studies was relatively low (0–9%) and the long silicosis latency and potential cessation of work on diagnosis meant that most or all total exposure occurs prior to disease—for example, in the study by Miller *et al* 5 and 15 participants had >2/1 and >1/1 radiographs on their last interim survey compared with 47 and 105 participants at follow-up.^11^ Furthermore, the alternative of using ORs for cross-sectional studies and hazard ratios for cohort studies presumes approximate equivalence; when disease prevalence increases significantly across the exposure range this is not the case.

To explore the population-level effect we calculated the absolute risk reduction (ARR). The Steenland study method was used to re-estimate the categorical cumulative silicosis risks for all studies.^14^ We then fitted non-parametric locally weighted least squares estimates (LOESS) and extracted the median risk equivalent of 40 years working at 0.1 mg/m^3^ (4 mg/m^3^-years). We used this to calculate the ARR when average exposure is reduced to 0.05 mg/m^3^ (2 mg/m^3^-years).

### Sensitivity analyses

We performed five sensitivity analyses: (1) we included only ILO major category 1 (or equivalent) silicosis; (2) we excluded cross-sectional studies; (3) we included exclusively miner cohorts; (4) we compared high (≥0.1 mg/m^3^) and low intensity studies among miners; and (5) to investigate heterogeneity at lower doses, we included only categories up to 8 mg/m^3^-years, the limit of model stability. Intensity (analysis 4) was our only pre-specified sensitivity analysis.

All codes and data are publicly available (https://github.com/pjhowlett/silica_drma).

## Results

Searches in Web of Science, Medline and Embase databases and citation searches yielded 1206 studies (figure 1). After de-duplication, 782 studies underwent title and abstract screening and 50 underwent full-text screening. Of 11 eligible studies, three reported overlapping cohorts and were excluded. Eight studies were therefore included with a total of 65 977 participants of whom 8792 had silicosis. The proportion with silicosis ranged between 4% and 32%. Agreement between reviewers was fair and substantial for title/abstract and full-text review, respectively (Cohen’s kappa 0.46 and 0.70).

### Characteristics of studies

Among the eight studies, five were cohort and three were cross-sectional studies (table 1). Chen *et al* reported on three cohorts, resulting in a total of 10 cohorts.^10^ Of these, six cohorts exclusively worked in the mining industry,^10 11 14 18 19^ two exclusively within industrial plants (pottery factory and foundry)^13 23^ and two consisted of workers from both, one with a predominance of miners (26 670/34 018, 78%)^25^ and the other with an undefined mix.^22^ Mean or median follow-up from initial exposure ranged from 24 to 37 years in six studies.^10 14 18 19 22 23^ In two other studies, minimum follow-up from first exposure was estimated at 13 years and 26 years, respectively.^11^ ^13^ Six studies used either ILO major category 1 radiographs or equivalent for diagnosis,^10 18 19 22 23 25^ one used death certificates in addition to ILO major category 1 radiographs^14^ and one used ILO major category 2 radiographs.^11^

**Table 1:** Characteristics of the seven studies which fit the inclusion criteria

Authors, year of publication,industry, location	Study duration and follow-up since first exposure	Exposure measurement	Diagnostic classification	Silicosis cases/sample size; risk	Analysis methods	Mean silicosis latency (years)	Mean intensity (mg/m^3^)	Loss to follow-up/ censoring
Cross-sectional studies								
Hnizdo and Sluis-Cremer, 1993Gold mines; South Africa^19^	Employed ≥10 years 1968–1971. Subsequent annual radiograph; post-employment follow-up to mean 1982 (range 1968–1991) via ‘occasional radiological examination’. Mean 24 years service (range 10–43)	Airborne shift dust sampling from 20 gold mines and 13 locations in mines by thermal precipitator and konimeter. Approximate silica content 30%^24 37^	ILO. Earliest radiograph ≥1/1*Blind reading by three trained readers; one who correlated best with autopsy chosen	313/2235 (14%)	Cumulative risk – SAS PROC LIFETEST (Life table) Cumulative risk model – SAS PROC LIFEREG; Loglogistic	34.6	0.087	0/2235 (0%)
Steenland and Brown, 1995Gold mines; USA^14^	Employed ≥1 year underground 1940–1965. Follow-up to 1990 via death certificate and radiographs during two cross-sectional surveys in 1960 and 1976. Average follow-up 37 years, average employment 9 years	1153 personal air samples with cyclone-filter. Silica via infrared spectrometry^38^	ILO. Earliest radiograph ≥1/1 or ‘small opacities’ and death certificate. Unclear radiology method	170/3330 (5%)128 cases by death certificate only, 29 by x-ray only, 13 by both	Cumulative risk – specified formula†	Not reported	0.0914% no data on exposure after 1975; possible underestimation	67/3330 (2%)
Hughes, *et al*1998Diatomaceous mine and processing; USA^22^	Employed >1 year 1942–1987. ‘Periodic radiographs’. Median employment 5.5 years (range 1–49.3 years). Median follow-up 29.9 years (range 1–53 years)^39^	5174 airborne shift samples from particle impinger and gravimetric pump. Silica content (10–25%) estimated based on materials processed^39 40^	ILO. Earliest radiograph ≥1/0 or large opacities. At least 2 of 3 certified ‘B’ readers for +ve result	81/1809 (4.5%)	Cumulative risk – SAS PROC LIFETEST (Life table) Cumulative risk model – SAS PROC LIFEREG; Loglogistic	11.4	1452/1809 had mean exposure <0.5 mg/m^3^357/1809 had mean exposure >0.5 mg/m^3^	Unclear. 214/2342 (9%) ‘unknown’ at study end in 1994^39^
Chen *et al*, 2005Tin mines, tungsten mines, pottery factories; China^10^	Started work after 1950, worked ≥1 year 1960–1974. Yearly radiographs since 1963. Follow-up to 1994. Minimum follow-up 26 years. Mean years of dust exposure pottery 24.9 years, tin 16.4 years, tungsten 16.5 years	Airborne gravimetric sampling from historical samples. Dust and silica content validated by side-by-side gravimetric sampling and X-ray diffraction	Chinese classification system. Earliest radiograph stage I or above. Agreement of 2 of 3 radiologists required	2816/14 427 (20%) tungsten miners,855/4028 (21%) tin miners, 785/4547 (17%) pottery workers	Cumulative risk – SAS PROC LIFETEST (Life table) Cumulative risk model – SAS PROC LIFEREG; Weibull	Tungsten: 19.0Tin: 20.2Pottery: 29.4	Tungsten: 0.2Tin: 0.152Pottery: 0.254	Tungsten 82/9007 (0.9%)Tin: 12/4028 (3%)Pottery: 4/4547 (0.1%)
Liu *et al*, 2013Metal mines, pottery factories; China^25^	Worked ≥1 year 1960–1974. All participants followed up to 2003 unless died or lost to follow-up. Yearly radiographs since 1963. Average follow-up 34.5 years	Airborne gravimetric sampling from historical samples. Dust and silica content validated by side-by-side gravimetric sampling and X-ray diffraction	Chinese classification system. Radiographs stage I or above. No radiology method reported	5297/34 018 (16%): 9007 tungsten miners 7663 iron miners 7348 pottery workers	No silicosis analytical methods	Not reported	Tungsten: 0.52Iron: 0.08Pottery:0.15–0.30 (1960–1980), 0.12–0.15 (post 1990)	1527/34 018 (4.5%)
Cross-sectional studies								
Kreiss and Zhen, 1996Hardrock mine;USA^18^	Random sample of dust exposed, aged >40 years in mining town. Radiographs performed in 1984 or 1986. Mean time from first exposure 36.1 years, minimum 13 years	Airborne samples; 649 job title-specific gravimetric dust measurements and 484 silica measurements between 1974 and 1982	ILO. Median of three B readers; radiologic profusion of >1/0	32/100 (32%)	No cumulative risk. Modelling: logistic regression	Not reported	Silicotics: 0.08Non-silicotics: 0.06	6/100 (6%) miners no exposure data
Miller *et al*, 1998Coal mine; Scotland^11^	Available radiographs from any years of 1970/1974/1978. Outcome based on follow-up radiograph in 1991. Minimum follow-up 13 years. Pit closure 1981	Regular (<yearly) airborne samples, initially thermal impinger then gravimetric pump. Silica via infrared spectrometry^41^	ILO. Most recent radiograph ≥2/1. Median result of three ‘experienced readers’	47/547 (9%)	No cumulative risk.Modelling: logistic regression	Not reported	Not reported	NA. However study is of 547/1416 (39%) traceable survivors
Rosenman, *et al* 1996Foundry; USA^23^	Employed ≥5 years before 1986; medical records up to 1991 used. Most recent radiographs obtained via work records or ‘other means’. Average follow-up 28.3 years and employment 19.2 years	Airborne samples; midget impinger. Silica conversion via bulk samples %	ILO. Most recent radiograph ≥1/0. At least 2 of 3 certified ‘B’ readers for +ve result	36/936 (4%)	No silicosis analytical methods	Not reported	0.23	NA

A valid correspondence between Chinese stage I, II, III and ILO 1, 2, 3 exists.^42^

* Autopsy performed in 131 miners with confirmation of silicosis in 128; 3 false positives.

† Cumulative risk (H(t)) = 1-− exp[-−sum of (hazards*interval width)]. All studies used job exposure matrices for personal exposure measurements. Study conversion factors were used where necessary.

ILO, International Labour Organisation

Average exposures varied within and across cohorts; all averages were above the current US PEL of 0.05 mg/m^3^. Steenland and Brown noted a decreasing intensity of exposure from 0.15 mg/m^3^ in cohorts hired before 1930 to 0.02 mg/m^3^ after 1950.^14^ Hughes *et al* show a differential dose-response among those exposed to intensities of <0.5 mg/m^3^ compared with >0.5 mg/m^3^, although this was confounded by higher exposures among early hires.^22^ A separate study of the Scottish Colliery cohort^11^ reported a small proportion of hours worked at intensities >1 mg/m^3^.^26^

### Risk of bias

Overall, the studies were deemed to have a low risk of bias (table 2 and online supplemental file 4). Most longitudinal studies reported frequent radiographs, particularly during employment; however, for one study only two radiographs were taken^14^ and in a second the frequency was unclear.^22^ In cohort studies, loss to follow-up ranged between 0% and 9%. Confirmation of baseline silicosis absence in two Chinese studies was not possible as mandatory chest radiographs were not introduced until 1963.^10 25^ Additional uncertainty due to conversion factors was reflected in the ascertainment of exposure scoring.^10 19 25^ A plot of log relative risks against cumulative exposures among miners (see online supplemental file 5) may suggest greater relative risks in studies with larger standard errors.

**Table 2:** Newcastle–Ottowa Scale (NOS) quality assessment tool

Study	Domains of point loss in NOS scale	Score out of 7
Cohort studies		
Hnizdo and Sluis-Cremer, 1993^19^	Exposure measure	6
Steenland and Brown, 1995^14^	–	7
Hughes *et al*, 1998^22^	–	7
Chen *et al*, 2005^10^	Exposure measure, lack of outcome at onset	5
Liu *et al*, 2013^25^	Exposure measure, lack of outcome at onset	5
Cross-sectional studies		
Kreiss and Zhen, 1996^18^	–	7
Rosenman, 1996^23^	Non-respondents/comparability	6
Miller *et al*, 1998^11^	–	7

Criteria are described in greater detail in online supplemental file 2 and 4.

### Cumulative risk

**Figure 3:**
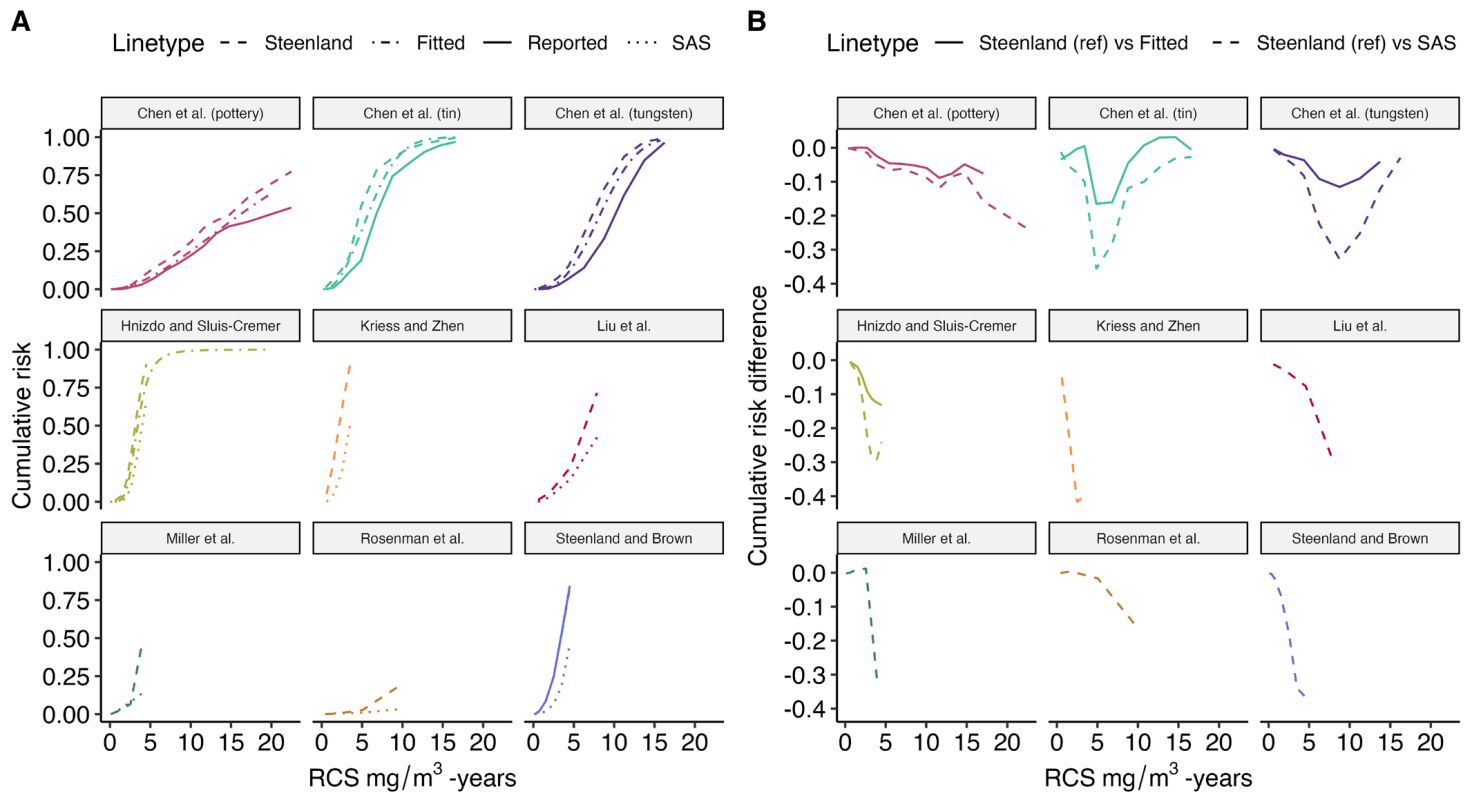
Comparison of calculated, reported and fitted cumulative risk of silicosis among studies providing categorical data (n=8). (A) Comparison of cumulative risks according to cumulative silica exposure for eight cohorts across six studies (NB: Hnizdo and Sluis-Cremer is represented by the full cohort and the selected group of only those who were active miners and attended an annual screening programme). (B) Re-representation of the data as the difference between the SAS LIFETEST formula and other methods according to cumulative silica exposure. This plot uses the same cohorts for A and B. These are represented by the same positioning and colour.

Re-estimated cumulative risk of silicosis (using the Steenland method) increased more rapidly and was larger in miner cohorts than in non-miner cohorts (figure 2A). In studies where cumulative exposures approached and exceeded 10 mg/m^3^-years there appeared to be a gradual flattening of the cumulative risk curve. Parametric fitted cumulative risk curves (figure 2B) show similar patterns. Among the parametric curves, the lowest risk is observed among diatomaceous earth industry workers who had low intensity exposure (<0.5 mg/m^3^); this cohort included an unknown proportion of miners and processing workers.^27^ Differences in cumulative risk estimates due to different methodology are clinically important, particularly at lower dose ranges of <10 mg/m^3^-years (figure 3A,B). Applied to all studies and cumulative dose categories compared with the Steenland method, the SAS LIFETEST method underestimated cumulative risk by a median of −7.2% (IQR −1.6% to −20.5%) while fitted risks underestimated cumulative risk by a median of −4.1% (IQR −0.4% to −8.2%).

### Dose-response meta-analysis

**Figure 4:**
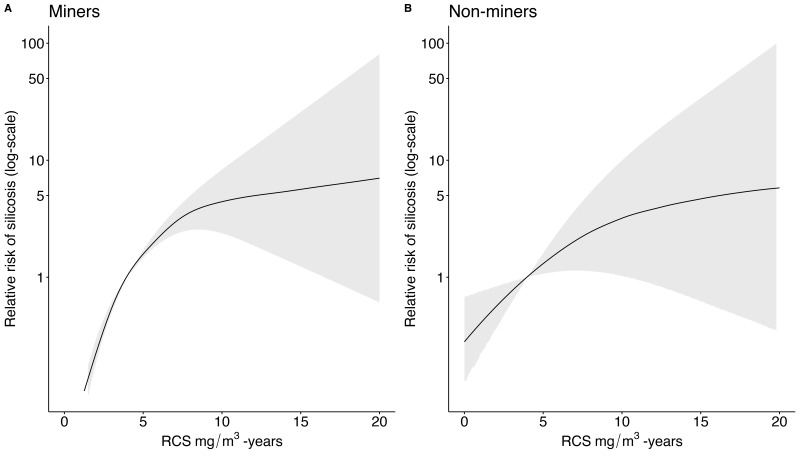
Dose-response meta-analysis of the relative risk of silicosis according to cumulative respirable cystalline silica (RCS) exposure. The reference category with a relative risk of 1 is fixed at 4 mg/m^3^-years, equivalent to 40 years working at 0.10 mg/m^3^. As the dose-response method would not allow for all persons in a category to achieve the outcome, 4 of the total 77 categories were combined. (A) Predicted increase in relative risk among mining cohorts (n=8 cohorts, providing 55 individual data points) using a restricted cubic spline model. Four knots at default quantiles were chosen (0.5, 0.35, 0.65 and 0.95), equivalent to 0.4, 2.0, 3.9 and 12.0 mg/m^3^-years. Heterogeneity was very high (I^2^=92.9%). (B) Predicted increase in relative risk among non-mining cohorts (n=2 cohorts, providing 18 individual data points). Three default knots were chosen (0.1, 0.5, 0.9), equivalent to 0.93, 6.2, and 15.4 mg/m^3^-years. Heterogeneity was high (I^2^=77.0%).

Among miners, the estimated relative risk reduction attributable to a reduction from 4 mg/m^3^-years silica exposure (equivalent to 40 years work at 0.1 mg/m^3^ intensity) to 2 mg/m^3^-years (equivalent to 40 years work at 0.05 mg/m^3^ intensity) is 0.23 (95% CI 0.18 to 0.29) (table 3). Heterogeneity was very high (I^2^=92.9%). Among non-miners, a similar reduction from 4 mg/m^3^-years to 2 mg/m^3^-years resulted in a relative risk of 0.55 (95% CI 0.36 to 0.83). Heterogeneity was high (I^2^=77.0%). Both relationships were non-linear (Wald test p<0.001). The dose-response meta-analysis fitted curves show a more rapid increase in relative risk of silicosis among miners than non-miners at cumulative doses of <10 mg/m^3^-years (figure 4). Compared with the primary analysis, none of the sensitivity analyses retaining the mining/non-mining stratification resulted in meaningful differences to the relative risk (see online supplemental file 6). Models stratified by exposure intensity resulted in larger relative risk estimates for low intensity cohorts. Restricting to exclusively miner cohorts and dose categories <8 mg/m^3^-years reduced the I^2^ to 74.1%. The reduced heterogeneity at lower doses can be visualised when log relative risks are plotted against cumulative exposures (see online supplemental file 5).

**Table 3:** Comparison of the relative risk and absolute risk reduction of silicosis at a cumulative respirable crystalline silica exposure of 2 mg/m^3^-years versus 4 mg/m^3^-years among miners and non-miners

	Risk ratio at reference4 mg/m^3^-years	Risk ratio at2 mg/m^3^-years (95% CI)	Absolute risk reduction of2 vs 4 mg/m^3^-years per1000 persons (95% CI)
Miners	1	0.23 (0.18 to 0.29)	323 (298 to 344)
Non-miners	1	0.55 (0.36 to 0.83)	23 (9 to 33)

For miners, the median estimated cumulative risk of silicosis from LOESS models at 4 mg/m^3^-years was 420 per 1000. If cumulative exposure was reduced to 2 mg/m^3^-years, the ARR was 323 (95% CI 298 to 344) fewer cases per 1000 miners. For non-miners, at 4 mg/m^3^-years the median estimate was 51 cases per 1000. If cumulative exposure was reduced to 2 mg/m^3^-years, this resulted in 23 (95% 9 to 33) fewer cases per 1000 non-miners. Data were only available from two non-mining studies, which demonstrate different cumulative risks with silicosis at 4 mg/m^3^-years (2.0% and 8.2%). Further LOESS cumulative risk estimates are shown in online supplemental file 7.

## Discussion

Previous narrative reviews have not rigorously quantified the relationship between cumulative silica exposure and silicosis risk. Our systematic review found the cumulative silicosis risk was higher among mining cohorts than non-mining cohorts at comparable cumulative silica exposure levels. Among mining cohorts, despite very high heterogeneity (I^2^=93%), we found a reduction from 4 to 2 mg/m^3^-years cumulative silica exposure was associated with a 77% (95% CI 71% to 82%) reduction in silicosis risk. This is equivalent to an average silica intensity reduction from 0.1 to 0.05 mg/m^3^ experienced over a 40-year average working lifetime. A recent systematic review suggests this level of average exposure reduction is achievable.^28^ Among non-mining studies, the reduction from 4 to 2 mg/m^3^-years was associated with a 45% (95% CI 34% to 67%) reduction in silicosis risk (I^2^=77%). These relative risk reductions result in meaningful absolute reductions. Among miners, the reduction from 4 to 2 mg/m^3^-years resulted in 323 (95% CI 298 to 344) fewer cases per 1000, while for non-miners this figure was 23 (95% CI 9 to 33) fewer cases per 1000.

Previous reviews used reported and fitted cumulative risk values directly from studies to infer disease burden at different exposure levels.^7 12^ Our study suggests these inferences may be imprecise because the cumulative risk methodology and underlying estimator assumptions varied across studies, resulting in clinically relevant differences in estimates. These differences are apparent at cumulative exposure values of <5 mg/m^3^-years, a highly relevant range in our review. Furthermore, a limitation of the LIFETEST method^10 19 20^ is the assumption that all intervals are equal. We used the Steenland method^14^ to account for uneven intervals.

One previous meta-analysis used a lifetable approach to calculate rate ratios for silicosis mortality^29^ and demonstrates a similar shape to our findings with values between those of miners and non-miners (see online supplemental file 8). This may be explained by four of the six studies including non-miners and a relatively fixed non-silicosis mortality quantity across categories. Our ARR values are in keeping with estimates from previous reviews outlined in the introduction.^7 12^

Our inclusion of two non-mining studies is too few to draw generalisable conclusions and is an oversimplification of the underlying diversity of industries. For example, a dose-response relationship has not been studied in current silicosis outbreaks with very high RCS exposures due to artificial stone^2^ and sand blasting.^30^ Choosing accelerated disease as an outcome or considering intensity as an exposure may provide early answers to clinically important questions. Other non-miner populations demonstrate a low incidence. For example, few cases were observed among Swedish and Chinese foundry workers^31 32^ and a similar relative risk profile to our study was observed among US industrial sand workers and German porcelain workers.^33 34^ One potential reason for differential responses between miner and some non-miner industries is the proportion of occluded (and thus biologically inactive) surfaces; when adjusted for the cumulative risk discrepancy between pottery and mining largely disappeared.^10^ Occlusion has also been demonstrated by aluminosilicates in coal mining.^35^ Alternative explanations include measurement error—that is, that (non-personal) airborne sampling in open workplaces overestimates true exposure. Importantly, given the range of industries, co-exposures and fatal recent outbreaks, caution is needed in drawing any conclusions until further (industry-specific) data are available.

Supporting previous assertions,^7 12^ we were able to quantify significant heterogeneity in our meta-analysis and dose-response analysis. We observed reduced but persistently high heterogeneity at lower dose ranges and in exclusively miner cohorts. Nevertheless, if caveated with the understanding that the variance in our estimates is almost exclusively due to between-study heterogeneity rather than statistical variability, we believe our meta-analysed estimates still provide clinically meaningful information. This is particularly true given the effect size, the importance of the policy question regarding chronic low exposures, the lack of alternative better methods and the absence of upcoming study data.

We observed an attenuation of the increase in the relative risk of silicosis among both miner and non-miner analyses at higher ranges of cumulative exposure. A clear discussion of the potential explanations is provided by Stayner *et al*.^36^ Of particular relevance are the healthy worker effect described in high exposure miners,^1^ the presence of a (yet undiscovered) resistant phenotype, misclassification of the exposure or outcome (for example, silica conversion errors have been suggested),^37^ saturation of biological disease pathways at higher silica exposures and potential modifying factors. Higher intensity exposure has been considered a potentiator for silicosis,^22 26^ but in our case this was not obviously apparent.

### Study limitations

Our study has important limitations. Most ILO cut-offs were 1/1 or 1/0. The absence of symptoms at lower ILO grades may limit the overall clinical impact estimated by the silicosis ARR, although further predicting symptomatic individuals at lower ILO grades is challenging.^5^ Due to unavailability of data we were unable to estimate ARRs at higher ILO thresholds. Importantly, in addition to silicosis, silica exposure has multiple often dose-response detrimental health effects; the silicosis ARR therefore represents the minimum potential health benefit of lowering permissible limits. Confounding and effect modification in the exposure-response relationship is possible: intensity,^22 26^ age,^14^ altitude^18^ and calendar-time^14 18 22^ have been suggested. However, none are conclusive and our univariate relationship remains a valid model. Substantial methodological differences in RCS measurements exist^10 37^ and there is a risk of measurement error. While this error may be random, it is potentially more pronounced in historical samples. Our grouping of miner and non-miner cohorts may incur misclassification; two miner cohorts included some non-miners, others potentially include non-mining job roles. The effect is unclear, although restriction to exclusively miner cohorts suggests similar effect sizes with marginal heterogeneity reduction. Despite stringent inclusion criteria requiring raw categorical data and prolonged follow-up, we were able to include most studies in previous reviews.^7 12^ Most studies had longer follow-up than our selection criteria and cohort loss to follow-up was low, limiting the concern of complete ascertainment of silicosis cases. However, selection bias due to loss to follow-up remains likely, particularly in the cross-sectional studies. The inclusion of different diagnostic criteria such as autopsy may introduce a differential bias, although our sensitivity analysis did not suggest this. Future research is needed to adjust for the effect of sub-radiological disease. The suggestion of increased relative risk among studies with large standard errors implies possible publication bias. However, with few studies over many decades and other potential explanations, it is unclear. Four full-text studies were not in English, online translation was not feasible and an important language bias exists. On reflection, our choice of the NOS is unlikely to capture the full extent of bias present in our studies. The results, particularly quantitative, should therefore be interpreted cautiously. While the assumptions required to choose ORs as the preferable unit of relative risk may introduce differential bias, we believe homogenising the unit of relative risk was the preferable option.

## Conclusion

Despite limitations, our study represents an important advance in answering a centuries-old question that remains globally relevant. Using reproducible and transparent methods, we have demonstrated clinically meaningful results that provide a strong argument for lowering RCS permissible exposure limits, particularly among miners, from 0.1 mg/m^3^ to 0.05 mg/m^3^. With supportive measures this change is possible. Further research among non-mining industries and in developing economies is warranted. Modern cohort study techniques mean prolonged follow-up is cheaper and easier and studies that associate carefully collected exposures to the risk of silicosis are feasible.

## supplementary material

10.1136/thorax-2024-221447 online supplemental file 1

## Data Availability

All data relevant to the study are included in the article or uploaded as supplementary information.
