# Supplementary material for: Relationship between cumulative silica exposure and silicosis: a systematic review and dose-response meta-analysis
Source: Thorax. 2024 Aug 6;79(10):e221447. doi: 10.1136/thorax-2024-221447 (PMC11503121; doi:10.1136/thorax-2024-221447)

The relationship between cumulative silica exposure and silicosis: A systematic review and dose-response meta-analysis

Supplementary material

Supplement 1. Full search strategy

We searched Web of Science, Medline and Embase for studies indexed prior to 24th February 2023. We included cohort and cross-sectional studies, with terms related to 'occupational exposure', 'silica' and 'silicosis'. No language restriction was added.

Web of Science

(TS=(silicos?s)) AND (TS=(Epidemiologic stud*) OR TS=(cohort stud*) OR TS=(Cohort analy*) OR TS=(Follow up stud*) OR TS=(observational stud*) OR TS=(Longitudinal) OR TS=(Retrospective) OR TS=(Cross sectional) OR TS=(clinical stud*)) AND (ALL=("Silicon dioxide") OR ALL=("quartz") OR ALL=("silica") OR ALL=("tridymite") OR ALL=("cristobalite") OR ALL=("SiO2") OR ALL=("coesite") OR ALL=("stishovite") OR ALL=("diatomaceous earth") OR ALL=("dust") OR ALL=("threshold limit value") OR ALL=("time weighted average") OR ALL=("occupational exposure"))

Medline

**Search Number**	**Term**
1	exp silicosis/
2	(Silicoses or silicosis).mp.
3	1 or 2
4	Clinical study/
5	Longitudinal study/
6	Retrospective study/
7	Prospective study/
8	Cohort analysis/
9	(Cohort adj (study or studies)).mp.
10	(follow up adj (study or studies)).tw.
11	(observational adj (study or studies)).tw.
12	(epidemiologic$ adj (study or studies)).tw.
13	(cross sectional adj (study or studies)).tw.
14	Cohort analy$.tw.
15	cross-sectional study/
16	4 or 5 or 6 or 7 or 8 or 9 or 10 or 11 or 12 or 13 or 14 or 15

17	occupational exposure.mp. or occupational exposure/ or (silica or quartz or tridymite or cristobalite or coesite or stishovite).mp. or silicon dioxide.mp. or exp silicon dioxide/ or diatomaceous earth.mp. or exp infusorial earth/ or *dust/ or maximum allowable concentration/ or "threshold limit value"/ or time weighted average.mp.
18	3 and 16 and 17

Embase

**Search Number**	**Term**
1	exp silicosis/
2	(Silicoses or silicosis).mp.
3	1 or 2
4	Clinical study/
5	Longitudinal study/
6	Retrospective study/
7	Prospective study/
8	Cohort analysis/
9	(Cohort adj (study or studies)).mp.
10	(follow up adj (study or studies)).tw.
11	(observational adj (study or studies)).tw.
12	(epidemiologic$ adj (study or studies)).tw.
13	(cross sectional adj (study or studies)).tw.
14	Cohort analy$.tw.
15	cross-sectional study/
16	4 or 5 or 6 or 7 or 8 or 9 or 10 or 11 or 12 or 13 or 14 or 15
17	occupational exposure.mp. or occupational exposure/ or (silica or quartz or tridymite or cristobalite or coesite or stishovite).mp. or silicon dioxide.mp. or exp silicon dioxide/ or diatomaceous earth.mp. or exp infusorial earth/ or *dust/ or maximum allowable concentration/ or "threshold limit value"/ or time weighted average.mp.
18	3 and 16 and 17

Supplement 2. Data extraction template

<u>Data extraction template</u>

General information

- Study ID
- Title
- Year of Publication
- Country in which the study was conducted
- Conflicts of interest

Characteristics of included studies

Methods

- Study design
    - Retrospective Cohort study
    - Prospective Cohort study
    - Cross sectional study
    - Other
- Study dates
- Radiographic diagnosis criteria
- Variables controlled for in analysis
- Statistical analysis performed
- Method of RCS concentration assessment
- Outcome Reported
    - Risk ratio
    - Prevalence
    - Silicosis cases/Exposed workers
    - Other

Participants

- Population description
- Inclusion criteria
- Exclusion criteria
- Sample Size
- Was there follow up after employment
- Worker Characteristics
    - Number of subjects
    - Age at first exposure
    - Total RCS concentration (mg/m^3)
    - Net years of exposure
    - Cumulative RCS exposure (mg/m^3-years)
    - Latency period (years)

    - Age at onset of silicosis
    - Age at last exposure
- Risk of Silicosis (stratified by each study's respective cumulative RCS exposure categories)
    - Cases
    - Workers entering category or Person-years
    - Silicosis Risk
    - Mean net exposure (years)
    - SD net exposure (years)
    - Mean latency period (years)
    - SD latency period (years)

<u>Quality assessment template</u>

Assessment of bias risk was based on the Newcastle Ottawa Scale for cohort and cross-sectional studies. Questions apply to both cohort and cross-sectional studies unless specified. Asterisks (*) indicate the number of points awarded for each option.

Selection 1: Representativeness of the exposed cohort
- a) Truly representative of the average worker in the community *
- b) Somewhat representative of the average worker in the community *
- c) Selected group of users e.g. workers who attend clinic
- d) No description of the derivation of the cohort

Selection 2: Selection of the non-exposed cohort
- a) Drawn from the same community as the exposed cohort *
- b) Drawn from a different source
- c) No description of the derivation of the non-exposed cohort

Note: our use of the word "cohort" referred to both the cohort (in cohort studies) and the sample (in cross-sectional studies)

Selection 3: Ascertainment of exposure
- a) Secure record (e.g. Record of Job History) **
- b) Structured interview *
- c) Written self report
- d) No description

Selection 4 (Cohort): Demonstration that outcome of interest (silicosis) was not present at start of study
- a) Yes *

- b) No

Selection 4 (Cross-sectional): Non-respondents

- a) Comparability between respondent and non-respondent characteristics is established, and the response rate is satisfactory. *
- b) The response rate is unsatisfactory, or the comparability between respondents and non-respondents is unsatisfactory
- c) No description of the response rate or the characteristics of the respondents and the non-respondents

Outcome 1 (Cohort): Assessment of outcome

- a) Independent blind assessment *
- b) Record linkage *
- c) Self report
- d) No description

Outcome 1 (Cross-sectional): Assessment of outcome

- a) Independent blind assessment **
- b) Record linkage **
- c) Self report
- d) No description

Outcome 2 (Cohort): Adequacy of follow up length for outcomes to occur

- a) Yes (Mean follow up ≥ 20 years from initial exposure) *
- b) No

Outcome 2 (Cross-sectional): Statistical test

- a) The statistical test used to analyse the data is clearly described and appropriate, and the measurement of the association is presented, including confidence intervals and the probability level (p value). *
- b) The statistical test is not appropriate, not described, or incomplete

Outcome 3 (Cohort): Adequacy of cohort follow up

- a) Complete follow up - all subjects accounted for *
- b) Subjects lost to follow up unlikely to introduce bias < 20% or description of those lost suggested no different from those followed *
- c) Follow up rate < 80%
- d) No statement

Supplement 3: Detailed description of two life table methods used for estimation of cumulative risk in the studies included in this review, the SAS LIFE TEST estimation used by Hnizdo et al and Chen et al, and the method by Steenland et al.

SAS LIFE TEST formula

The formula used by Hnizdo et al and Chen et al references the SAS LIFE TEST formula(1–3). The results in the respective papers can be reproduced through the following calculations, with reference to the SAS LIFE TEST manual.

The papers use the term "cumulative risk" to refer to the cumulative distribution function $F(a) = 1 - S(a_i)$, where $S(a)$ is the survival function (defined pg.5120 in SAS manual). $F(a)$ can be interpreted as the cumulative probability that a person will develop silicosis by a certain level of exposure, a. In a silicosis life table, $S(a_i)$ can be estimated as the product of the estimated survival in the previous concentration interval ($S(a_{i-1})$) and one minus the conditional probability of an event in the interval $[a_{i-1}, a_i)$, estimated by number of cases in the interval divided by the effective sample size of the interval. Given d_i silicosis cases in the exposure interval $[a_{i-1}, a_i)$ and n_i individuals entering the category, n'_i represents the effective sample size of the interval (assuming censoring is uniformly distributed over the interval) and is $n'_i = n_i - w_i/2$ where w_i is the number of individuals censored in the interval.

The survival function may then be estimated by:

$$\hat{S}(a_i) = \begin{cases} 1 & i = 0 \\ \hat{S}(a_{i-1})p_{i-1} & i > 0 \end{cases}$$

where $p_{i-1} = 1 - \frac{d_{i-1}}{n'_{i-1}}$

A worked example, for the Tungsten cohort in Chen et al, is shown here. In this case, the 'at risk' figure provided in the paper represents the effective sample size (n'_i) of the interval:

Cohort	RCS dose interval (mg/m3-yrs)	d_i	n'_i	d_i/n'_i	$p_i = 1- d_i/n'_i$	$S(i)$	$F(i) = 1- S(i)$	Reported risk
Chen et al. (tungsten)	0, <1.24	53	11686	0.00	1.00	1.00	**0.00**	**0.00**
Chen et al. (tungsten)	1.25, <2.5	185	7625	0.02	0.98	1.00	**0.00**	**0.01**

Chen et al. (tungsten)	2.5, <3.75	244	5560	0.04	0.96	0.97	**0.03**	**0.03**
Chen et al. (tungsten)	3.75, <5.00	320	4325	0.07	0.93	0.93	**0.07**	**0.07**
Chen et al. (tungsten)	5.00, <7.5	708	3179	0.22	0.78	0.86	**0.14**	**0.14**
Chen et al. (tungsten)	7.5, <10.00	773	1791	0.43	0.57	0.67	**0.33**	**0.33**
Chen et al. (tungsten)	10.00, <12.5	411	686	0.60	0.40	0.38	**0.62**	**0.62**
Chen et al. (tungsten)	12.5, <15.0	114	149	0.77	0.23	0.15	**0.85**	**0.85**
Chen et al. (tungsten)	15.00, <17.5	8	10	0.80	0.20	0.04	**0.96**	**0.96**

Steenland et al formula

Steenland et al. work with a discrete hazard function assuming constant hazard in interval and estimate the cumulative hazard from that, to allow for unequal length exposure intervals(4,5). The hazard rate in each exposure interval may be estimated as:

$h(a_i) = d_i / (b_i * n'_i)$

Where b_i is the width of the exposure interval $[a_{i-1}, a_i)$. In the case of a constant event rate (λ) the hazard at exposure level a is

$h(a) = \lambda$

with cumulative hazard up to exposure level a estimated as

$H(a) = \lambda a$

Using the relationship between cumulative hazard and the survival function, 1 – S(a) can be estimated as

$1 - S(a) = 1 - e^{-\lambda a}$ (in Kirkwood and Sterne pg 283)

In the case of categorical risks, λa is equal to the sum of the category hazards multiplied by their respective interval widths, and the formula below – as used in Steenland et al – may be used:

$F(a) = 1 - e^{-\Sigma \lambda_i a_i}$

A worked example, for Steenland et al. The effective sample size (n'_i) is calculated by the number entering the category – cases/2:

Cohort	RCS dose interval (mg/m3-yrs)	d_i	n'_i	b_i	$h(t_i)$	$\Sigma \lambda_i t_i$	$F(i) = 1- S(i)$	Reported (unadjusted) risk
Steenland and Brown	0 , <0.2	5	3327.5	0.20	0.01	0.00	**0.00**	**0.00**
Steenland and Brown	0.2, <0.5	5	1797.5	0.30	0.01	0.01	**0.01**	**0.01**
Steenland and Brown	0.5, <1.0	15	1052.5	0.50	0.03	0.02	**0.02**	**0.02**
Steenland and Brown	1.0, <2.0	33	667.5	1.00	0.07	0.09	**0.08**	**0.08**
Steenland and Brown	2.0, <3.0	44	309	1.00	0.19	0.28	**0.24**	**0.25**
Steenland and Brown	3.0, <4.0	42	104	1.00	0.47	0.76	**0.53**	**0.53**
Steenland and Brown	4.0	26	39	1.50	0.67	1.76	**0.83**	**0.84**

Bibliography

1. Chen W, Hnizdo E, Chen JQ, Attfield MD, Gao P, Hearl F, et al. Risk of silicosis in cohorts of Chinese tin and tungsten miners, and pottery workers (I): An epidemiological study. Am J Ind Med. 2005 Jul;48(1):1–9.

2. Hnizdo E, Sluis-Cremer GK. Risk of silicosis in a cohort of white South African gold miners. Am J Ind Med. 1993 Oct;24(4):447–57.

3. SAS Institute Inc. The LIFETEST Procedure. In: SAS/STAT® 141 User'sGuide [Internet]. Cary, NC, USA; 2015. Available from: https://support.sas.com/documentation/onlinedoc/stat/141/lifetest.pdf

4. Steenland K, Brown D. Silicosis among gold miners: exposure--response analyses and risk assessment. Am J Public Health. 1995 Oct;85(10):1372–7.

5. Kirkwood BR, Sterne JAC, Kirkwood BR. Essential medical statistics. 2nd ed. Malden, Mass: Blackwell Science; 2003. 501 p.

6. Mannetje A, Steenland K, Attfield M, Boffetta P, Checkoway H, DeKlerk N, et al. Exposure-response analysis and risk assessment for silica and silicosis mortality in a pooled analysis of six cohorts. Occup Environ Med. 2002 Nov;59(11):723–8.

Supplement 4. Quality assessment tool

Maximum points:	Selection					Outcome					Total (7 points)
	Representative ★	Exposure selection ★	Exposure measure ★	Lack of outcome at onset ★	Non-respondents /comparable ★	Outcome measure ★	Outcome measure ★★	Duration ★	Statistical test ★	Follow up ★	
Cohort Studies											
Hnizdo and Sluis-Cremer, 1993	★	★	-	★	n/a	★	n/a	★	n/a	★	6
Steenland and Brown, 1995	★	★	★	★	n/a	★	n/a	★	n/a	★	7
Hughes et al., 1998	★	★	★	★	n/a	★	n/a	★	n/a	★	7
Chen et al., 2005	★	★	-	-	n/a	★	n/a	★	n/a	★	5
Liu et al., 2013	★	★	-	-	n/a	★	n/a	★	n/a	★	5
Cross-sectional Studies											
Kreiss and Zhen, 1996	★	★	★	n/a	★	n/a	★★	n/a	★	n/a	7
Rosenman, 1996	★	★	★	n/a	-	n/a	★★	n/a	★	n/a	6
Miller et al., 1998	★	★	★	n/a	★	n/a	★★	n/a	★	n/a	7

Supplement 5. Log relative risk of silicosis according cumulative silica exposure, separated by study cohort. The size of the point is relative to the standard error of the estimate. For both cases, the baseline cumulative dose is not standardised. Plot A represents miner cohorts. Plot B represents non-miner cohorts. The rapid initial increase in log relative risk in Plot B is explained by the baseline group having only 2 cases, a much lower number than all groups. If the two lowest exposure groups are combined, the relative risk profiles of the two studies can be observed to become more similar.

A

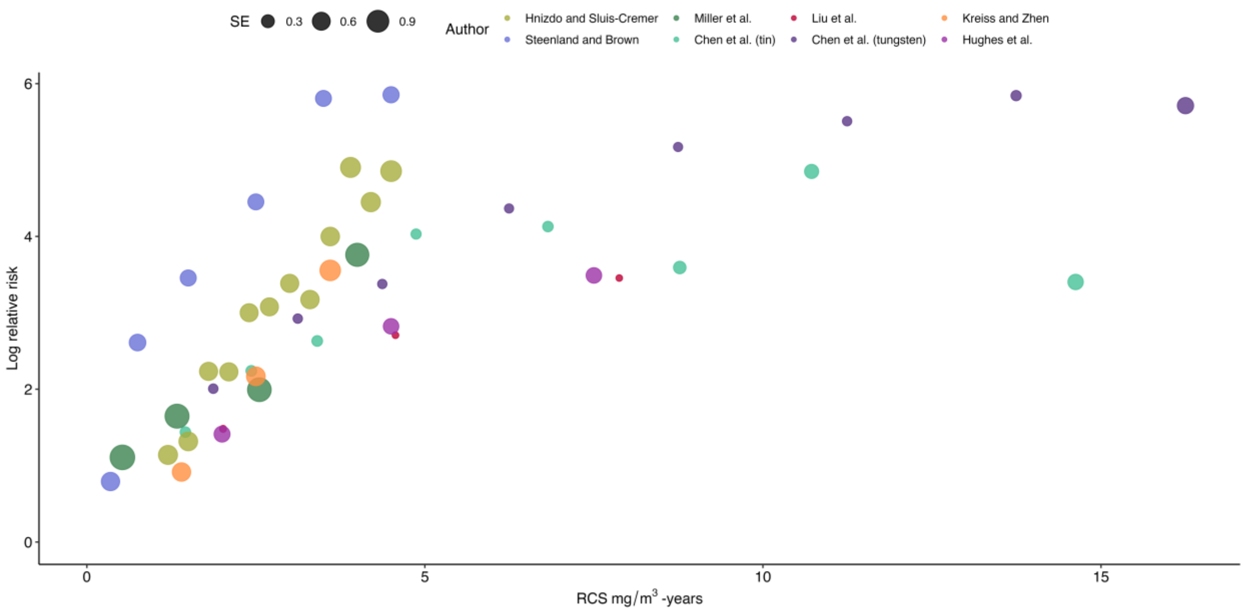

B

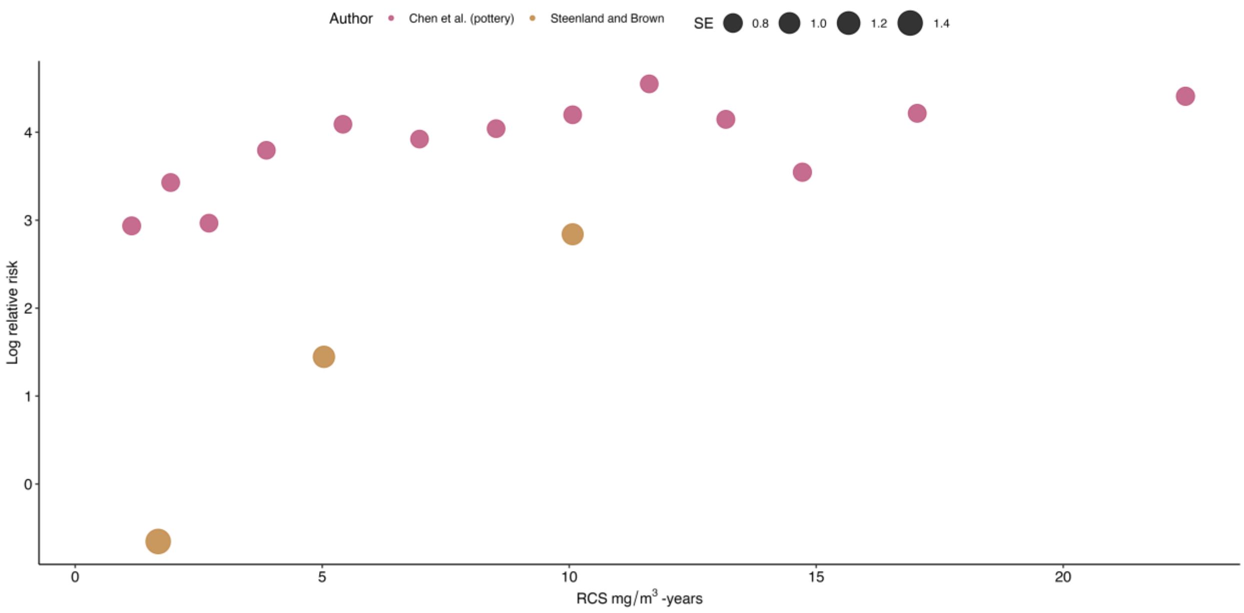

Supplement 6. Table of relative risks of silicosis at cumulative exposures of 1, 2, 4, 10 mg/m^3-years for the full cohorts compared to sensitivity analyses, calculated by dose-response meta-analysis using previously described methods.

	Subgroup	1 mg/m^3-years	2 mg/m^3-years	4 mg/m^3-years (reference)	10 mg/m^3-years	Heterogeneity (I^2)
All cohorts	Miners	0.07 (0.05 – 0.10)	0.23 (0.18 – 0.29)	1	4.45 (2.38 – 8.32)	92.9%
	Non-miners	0.40 (0.22 – 0.75)	0.55 (0.36 – 0.83)	1	3.20 (1.03 – 9.95)	77.0%
Sensitivity analysis						
Only major category 1 / Only cohort studies	Miners	0.07 (0.05 – 0.11)	0.23 (0.17 – 0.31)	1	4.14 (2.10 – 6.84)	93.7%
	Non-miners	-	-	-	-	-
Exclusively miner cohort	Miners	0.06 (0.04 – 0.08)	0.21 (0.17 – 0.24)	1	3.57 (1.49 – 9.19)	87.3%
Stratified by intensity	High intensity (≥0.1)	0.08 (0.07 – 0.09)	0.22 (0.21 – 0.24)	1	3.39 (1.37 – 8.39)	88.6%
	Low intensity (<0.1)	0.03 (0.02 – 0.06)	0.17 (0.12 – 0.25)	1	41.37 (0.86 – 1986.84)	0.0%
Under 8 mg/m^3-years	Miners	0.07 (0.05 – 0.10)	0.24 (0.19 – 0.32)	1	-	89.0%
	Exclusively miner	0.06 (0.04 – 0.09)	0.23 (0.17 – 0.30)	1	-	74.1%

Supplement 7. Predicted cumulative risk of participants with silicosis according to different cumulative RCS exposures. Based on locally weighted least squares estimates (LOESS) of the proportion of miners with silicosis at selected cumulative RCS exposures. Note for the calculation of a median estimate at 4 mg/m^3, the study by Kriess and Zhen was not used as it did not contribute to both 2 and 4 mg/m^3-years groups.

Study	Population	Cumulative risk (%)				
		1 mg/m^3-yrs	2 mg/m^3-yrs	4 mg/m^3-yrs	10 mg/m^3-yrs	20 mg/m^3-yrs
Miners						
Chen et al. (tin)	Tin miners	2.5	12.4	36.7	91.4	-
Chen et al. (tungsten)	Tungsten miners	1.1	3.0	13.1	77.3	-
Hnizdo and Sluis-Cremer (select)	Gold miners	0.3	6.5	75.7	-	-
Miller et al.	Coal miners	2.1	5.0	47.3	-	-
Kreiss and Zhen	Hardrock miners	14.9	47.0	-	-	-
Mixed miners/non-miners						
Liu et al.	Metal mines/ pottery factories	2.4	6.0	19.5	-	-
Non-miners						
Chen et al. (pottery)	Pottery factory	0.4	2.6	8.2	31.8	69.5
Rosenman et al.	Foundry workers	0.2	0.3	2.0	20.1	-

Supplement 8. Plot of miner and non-miner predicted relative risks of silicosis morbidity/disease and the meta-analysed rate ratios for silicosis mortality (here presented as relative risks) according to Mannet'je et al, at the dose categories represented in the study(6)

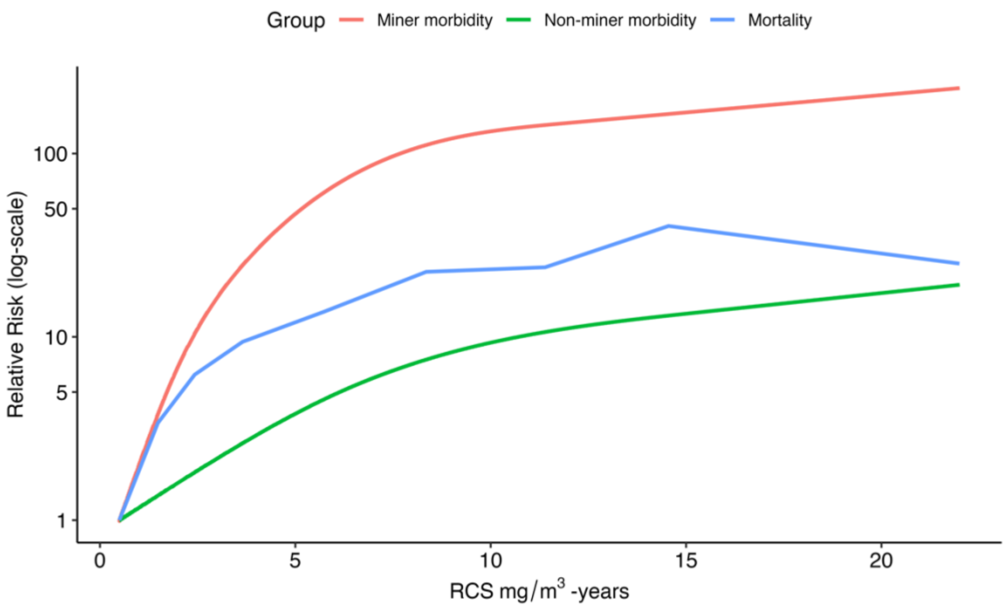

Supplement: online supplemental file 1 [file thorax-79-10-s001.pdf]
